# Fusing hand-crafted and deep-learning features in a convolutional neural network model to identify prostate cancer in pathology images

**DOI:** 10.3389/fonc.2022.994950

**Published:** 2022-09-27

**Authors:** Xinrui Huang, Zhaotong Li, Minghui Zhang, Song Gao

**Affiliations:** ^1^ Department of Biochemistry and Biophysics, School of Basic Medical Sciences, Peking University, Beijing, China; ^2^ Institute of Medical Technology, Health Science Center, Peking University, Beijing, China; ^3^ Department of Pathology, Guangdong Provincial People’s Hospital, Guangzhou, China

**Keywords:** prostate cancer, pathology image, convolutional neural network model, feature fusion, classification

## Abstract

Prostate cancer can be diagnosed by prostate biopsy using transectal ultrasound guidance. The high number of pathology images from biopsy tissues is a burden on pathologists, and analysis is subjective and susceptible to inter-rater variability. The use of machine learning techniques could make prostate histopathology diagnostics more precise, consistent, and efficient overall. This paper presents a new classification fusion network model that was created by fusing eight advanced image features: seven hand-crafted features and one deep-learning feature. These features are the scale-invariant feature transform (SIFT), speeded up robust feature (SURF), oriented features from accelerated segment test (FAST) and rotated binary robust independent elementary features (BRIEF) (ORB) of local features, shape and texture features of the cell nuclei, the histogram of oriented gradients (HOG) feature of the cavities, a color feature, and a convolution deep-learning feature. Matching, integrated, and fusion networks are the three essential components of the proposed deep-learning network. The integrated network consists of both a backbone and an additional network. When classifying 1100 prostate pathology images using this fusion network with different backbones (ResNet-18/50, VGG-11/16, and DenseNet-121/201), we discovered that the proposed model with the ResNet-18 backbone achieved the best performance in terms of the accuracy (95.54%), specificity (93.64%), and sensitivity (97.27%) as well as the area under the receiver operating characteristic curve (98.34%). However, each of the assessment criteria for these separate features had a value lower than 90%, which demonstrates that the suggested model combines differently derived characteristics in an effective manner. Moreover, a Grad-CAM++ heatmap was used to observe the differences between the proposed model and ResNet-18 in terms of the regions of interest. This map showed that the proposed model was better at focusing on cancerous cells than ResNet-18. Hence, the proposed classification fusion network, which combines hand-crafted features and a deep-learning feature, is useful for computer-aided diagnoses based on pathology images of prostate cancer. Because of the similarities in the feature engineering and deep learning for different types of pathology images, the proposed method could be used for other pathology images, such as those of breast, thyroid cancer.

## 1 Introduction

Prostate cancer, the most malignant cancer that occurs in men, follows lung cancer in males in terms of fatality rates (1). Transrectal ultrasound guided prostate biopsy is commonly considered the “golden standard” of available examinations such as rectal examinations, ultrasound examinations, X-ray imaging, and serum immunology examinations. These examination types directly determine the benign or malignant characteristic of the prostate and influence the patient treatment plan (e.g., radiotherapy, chemotherapy, resection, or conservative treatment). However, analyzing the large number of pathology images from biopsy tissues for treatment decision-making is a significant burden on pathologists. In recent years, radiomics has developed rapidly with the extraction of quantitative metrics—the so-called radiomic features—within medical images to capture tissue and lesion characteristics such as heterogeneity and shape and may, alone or in combination with demographic, histologic, genomic, or proteomic data, be used for clinical problem solving (2). For example, using machine learning (ML) techniques, the information from pathology images can be extracted by automatically extracting quantitative pathological features for high-throughput judgment (3–6), which has the potential to increase the accuracy, consistency, and reliability of prostate cancer diagnosis using histopathology. There are two types of features in the field of ML: hand-crafted features based on traditional ML and learned features based on deep learning (DL) (7–9). The most significant difference between the features of traditional ML and DL is that the former are manually designed, whereas the latter are automatically extracted by a convolutional neural network (CNN) (10). Hand-crafted features were designed based on the expertise of pathologists with respect to clinical pathology diagnoses. For example, Sertel et al. extracted color–texture characteristics from a model-based intermediate representation to propose an automated grading method for the quantitative analysis of the histopathology images of follicular lymphoma (11). However, in 2018, a comprehensive review highlighted several problems associated with the application of ML to histopathology image analysis, which include large image sizes, insufficiently labeled images, information magnification of different levels, unordered-texture images, and color variation and artifacts (12). DL based on a CNN can overcome several of the abovementioned limitations and improve the ability to identify subtle differences in histopathological characteristics, thus allowing computers to “see” new features or identify weak signals in images. For example, Coudray et al. trained a deep CNN (Inception v3) on whole-slide images obtained from The Cancer Genome Atlas to accurately and automatically classify them as lung adenocarcinoma, lung squamous cell carcinoma, or normal lung tissue (13). However, the lack of interpretability hinders the clinical application of DL (14).

As described above, there are advantages and disadvantages to the features used in traditional ML and DL; thus, the effective fusion of these two types of features is critical to the further performance improvement of computer-aided diagnosis (15), as has been found in medical radiology image analysis. For example, a classification model of benign and malignant breast cancer extracted low- to mid-level VGG19 features as well as hand-crafted radiology features including size, shape, texture, and morphological features from mammography, ultrasound, and magnetic resonance images (16). In addition, a method for the automated classification of lung nodules on chest computerized tomography was presented to distinguish benign and malignant nodules by fusing texture, shape, and DL information (17). A pathology image classification algorithm for malignant and benign skin tumors combines the DL feature extraction from an encoding CNN and traditional features, including texture and color features extracted from a gray-level co-occurrence matrix (GLCM) (18) and the color moment (19), respectively. The core of this algorithm is a feature fusion algorithm that can automatically adjust the proportion of high-level DL features and traditional features (20). Similarly, the fusion of digital histopathology and Raman chemical imaging modalities has the potential to improve the binary classification of prostate cancer pathology images by integrating both morphological and biochemical information across data sources (21). Most recently, to mine more information from different radiomics data in multicenter studies and identify personalized predictive and/or prognostic models to improve the reproducibility, an image biomarker standardization initiative (IBSI) was introduced for the standardization of radiomic features (22), and some radiomics computational frameworks were developed to comply IBSI and allow users to complete the whole radiomics workflow within the same software, simplifying the radiomics process, such as the matRadiomics software (23).

In this paper, we propose a classification fusion network for prostate pathology images, which fuses eight advanced features, namely, seven hand-crafted features and one DL feature. The hand-crafted features are the local features of the scale-invariant feature transform (SIFT) (24), speeded up robust feature (SURF) (25), and oriented features from accelerated segment test (FAST) and rotated binary robust independent elementary features (BRIEF) (ORB) (26), cavity histogram-of-oriented-gradients (HOG) (27) features, cell nuclei texture and shape features, and dye color features. The DL feature is obtained from the convolutional output layer of a ResNet-18. A model was created to effectively combine these different features. This model consists of three parts: a matching network to ensure that the different dimensions of each feature are consistent, an integrated network to ensure that features are represented in a sparse manner, and a fusion network to output the classification result.

The results of the experiments and multiple analyses reveal that the proposed method can effectively determine the difference between positive and negative images.

## 2 Materials and methods

### 2.1 Data collection

The pathology images of the prostate were obtained from hematoxylin and eosin slides at 40× magnification with Olympus microscopes (Olympus, BX53) at the Department of Pathology, Health Science Center, Peking University, China, and the Department of Pathology, Guangdong Provincial People’s Hospital, Guangzhou, China. The image size was 680 × 512 pixels and 744 × 512 pixels respectively. Based on their histologic features, two or three expert pathologists diagnosed any diseases that appeared in these images.

Finally, 1100 high-quality prostate pathology images with typical features were used in the study, 546 of which showed signs of prostate cancer.

### 2.2 Hand-crafted features

#### 2.2.1 Qualitative diagnosis

In general, the pathologists qualitatively diagnosed the prostate pathology images based on the following features (28, 29).

The normal prostate tissue contains glands (including a gland cavity and bilayer columnar epithelial cells), stroma, nuclei, stones, and other anatomical structures. This tissue can be divided into four characteristic structures: 1) the large gland cavity structure, where the gland cavity is large and the papillary protrusion causes the gland cavity to exhibit a plum-like structure; 2) the leaf structure, where the gland cavity and the gland lobe are divided into lobes; 3) the amyloid bodies in the gland cavity; and 4) a bilayer structure consisting of the inner and outer epithelial cells around the gland cavity. Malignant prostate tissue does not generally exhibit these structural characteristics.

There are three important indicators for the diagnosis of prostate cancer: 1) Cell heterogeneity: In general, the nucleus area of cancerous cells is larger than that of normal cells, and the variance in the nucleus sizes in the images is substantial. Moreover, chromatin in cancer cells is clumped on the side, and its nuclear membrane is clear. A large nucleolus with a diameter greater than 1.2 μm is a significant diagnostic indicator of prostate cancer. 2) Invasion phenomenon: Multiple small gland cavities are present in prostate cancer tissue, which directly leads to changes in the average size and variance of the gland cavity area. The boundary of the gland cavity in prostate cancer tissue is smooth without the papillary protrusions found in normal prostate tissue. 3) Disordered tissue structure: The normal prostate gland is centered on the urethra, radially distributed around it, and the inner edges of the acini are undulating and serrated. In prostate cancer, the regular growth shape of the gland disappears and the acini grow irregularly in all directions, which directly changes the texture characteristics.

#### 2.2.2 Quantitative parameters

Feature engineering is a key phase in the design of pathology image classification methods that impacts the final classification outcome (30). Based on the qualitative diagnosis characteristics described by the pathologists, seven hand-crafted features were extracted from the prostate pathology images: the texture and shape features of the cell nuclei; the gradient features of the cavity; the local image features; and the color features of the dye. Specifically, the GLCM was calculated to describe the texture; the Fourier descriptor (31) was selected to describe the shape; HOG was used to determine the gradient characteristic of the cavity; and the SIFT, SURF, and ORB were used to describe the local image features. Moreover, the color correlogram (32), which eliminates the influence of different stains, was selected to describe the color.

##### 2.2.2.1 Texture and shape features of the cell nucleus

To describe the texture of a cell nucleus, the GLCM was calculated. The GLCM describes the texture information of the image according to the probability of the repeated occurrence of the gray-scale structure. In particular, it describes the number of times that pixel pairs with distance *d* and direction *θ* (0°, 45°, 90°, and 135°) occur. Several statistical properties of texture features calculated from the GLCM indirectly reflect the image texture characteristics, which include the energy, dissimilarity, contrast, homogeneity, and correlation. Finally, we employed a six-dimensional feature vector to represent the image texture features.

To describe the shape features of a cell nucleus, the Fourier descriptor was used to describe the contour of the object. The Fourier descriptor converts the contour feature from the spatial domain to the frequency domain using the Fourier transform of the object boundaries in an image. In this study, we extracted the first 60 dimensions of the frequency domain as the feature vectors of the image. For vectors with fewer than 60 dimensions, we padded the ends of the 60-dimensional vector with the high-frequency information after shifting the zero-frequency component to the center of the spectrum.

##### 2.2.2.2 Gradient features of the gland cavity

The HOG was employed to represent the cavity features, given that the number of cavities between the cells in a pathology image is significantly less than those in the cells. The HOG is a feature descriptor used for object detection in computer vision and image processing that represents the gradient information in different directions within the local regions of an image. In this study, we modified the HOG descriptor to count the cavity features between the cells.

##### 2.2.2.3 Local features described with SIFT, SURF, and ORB

SIFT, SURF, and ORB are three common methods used to describe the local feature points in an image.

These image local feature points are generally employed to match and recognize images. They can reveal the most important parts of an image and do not change when the image’s brightness, shape, or noise levels change, e.g., the points of corners and edges or the highlights and dark spots.

##### 2.2.2.4 Color features

The color correlogram is a representation of the image color distribution that indicates the proportion of pixels of a certain color with respect to the entire image and reflects the spatial correlation between different color pairs. Experimental results have revealed that color correlograms perform retrieval more efficiently than color histograms and color coherence vectors. It is complex to compute the color correlogram with respect to the correlation between any colors. By contrast, the color auto-correlogram is a simple scheme that only shows how pixels of the same color are related to each other in space. For efficiency and convenience, we used the color auto-correlogram to extract the color features. In addition, the color auto-correlogram can eliminate the image differences caused by different staining owing to its consideration of the statistical properties of the same color.

### 2.3 DL feature

DL features were extracted from the convolutional and fully connected layers of the CNN. These features include abstract visual characteristics. Most researchers agree that spatially closer pixels are more connected than distant ones. Each neuron requires only local visual information, not the full image. Then, global information is compiled from the local data.

### 2.4 Fusion CNN construction

Although the hand-crafted and DL features contain a wide range of information that is important for the global pathology slice in addition to the local tissue, we still need an effective method to aggregate these features from various scales to increase the overall accuracy, which should be higher than the values corresponding to any individual feature.

**Figure 1:**
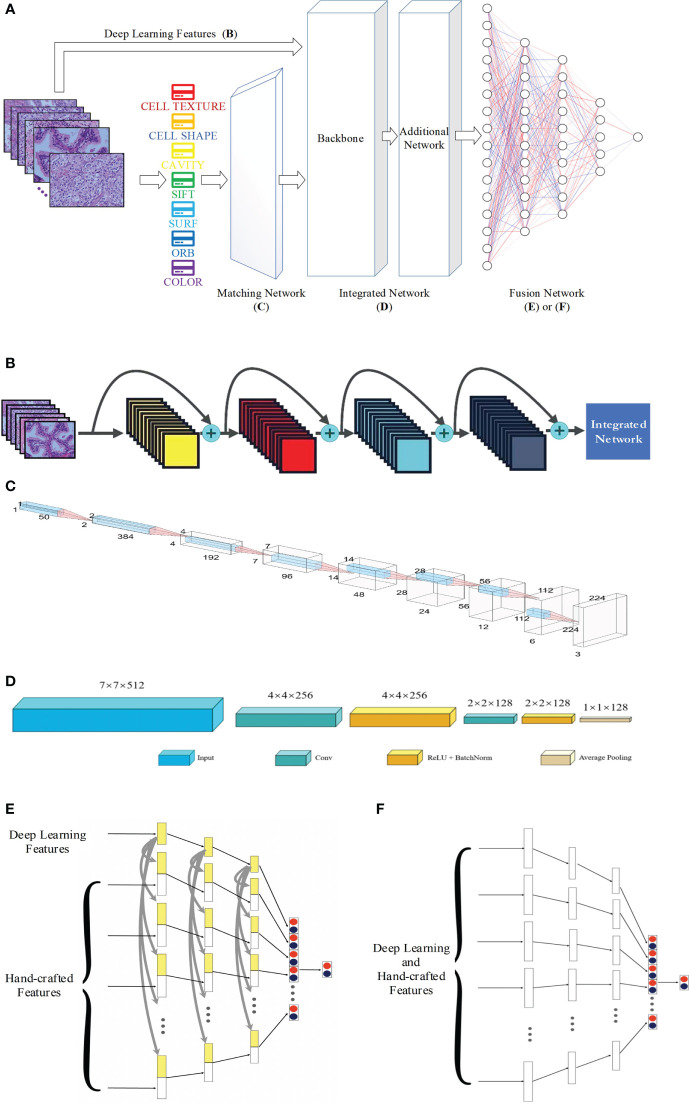
Proposed network for pathology image classification: **(A)** the whole workflow; **(B)** DL feature extraction; **(C)** architecture of the matching network; **(D)** architecture of the integrated network; **(E)** and **(F)** for architecture of the fusion network with and without concatenation.

**Figure 1A** shows that, apart from hand-crafted feature and DL feature extraction (**Figure 1B**), the proposed network is made up of three components: the matching network (**Figure 1C**), which is used to adjust the hand-crafted features; the integrated network (**Figure 1D**), which is used to process all of the features; and the fusion network (**Figures 1E**, **F**), which is used to combine different features. In the following sections, further details will be provided regarding each network.

#### 2.4.1 Feature extraction

##### 2.4.1.1 Hand-crafted feature extraction

**Figure 2:**
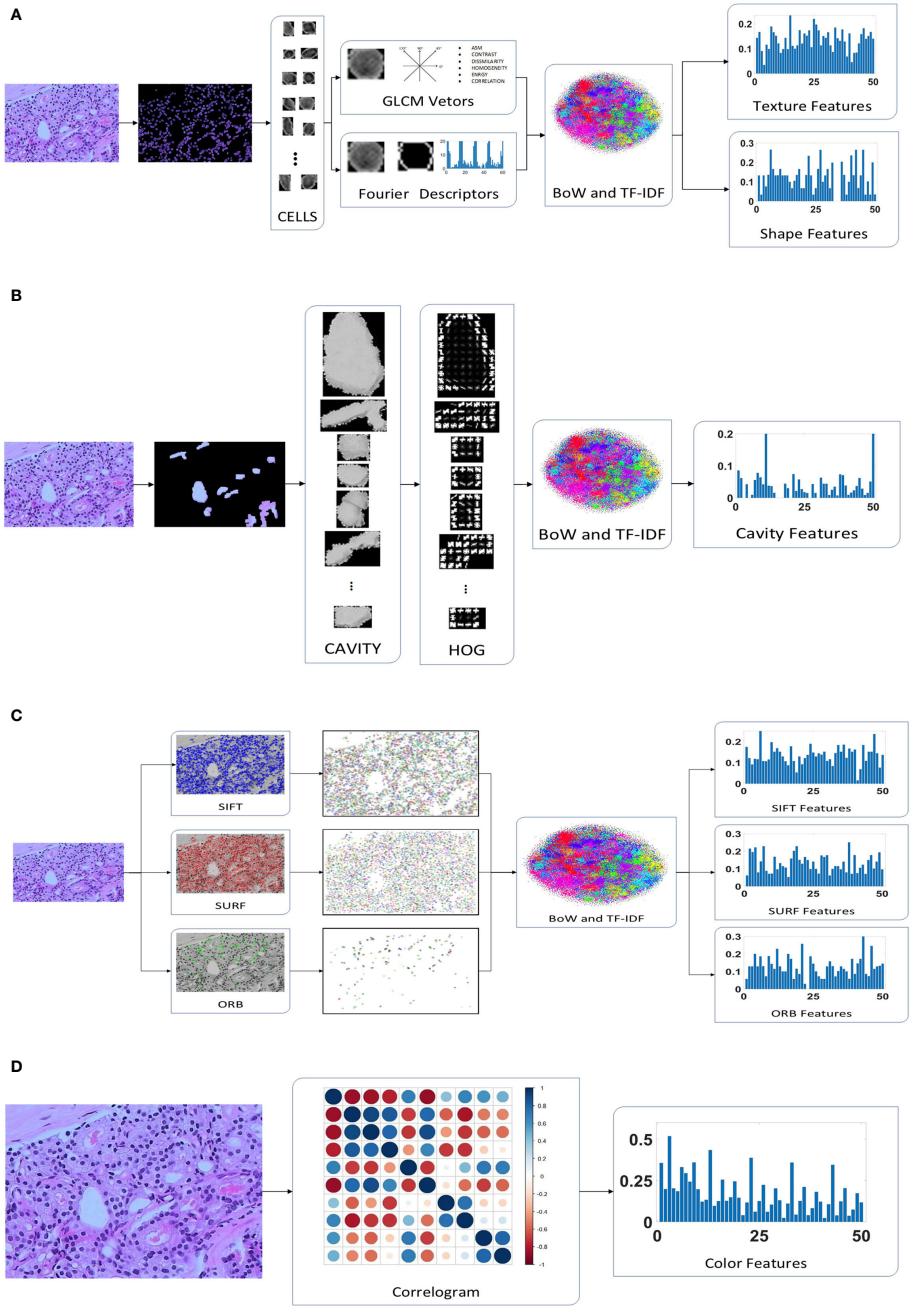
Hand-crafted feature extraction: **(A)** texture and shape feature extraction of the cell nuclei; **(B)** cavity feature extraction; **(C)** feature extraction; **(D)** color feature extraction.

**Figure 2** presents the extraction process of the hand-crafted features. Cell nucleus characteristics that include the texture and shape features shows in **Figure 2A**. To obtain the texture feature, the critical step is the calculation of the GLCM. First, all cell nuclei are extracted from the original pathology images using the watershed segmentation algorithm. Thereafter, the six statistical properties calculated from the GLCM of each cell nucleus image are used to form the texture features of the corresponding nucleus. Finally, we input all the texture features of the nucleus to the combined bag of words (BoW) (33) and term frequency–inverse document frequency (TF–IDF) (34) model (as detailed in Section 2.4.2) to obtain the texture features of each pathology image. Similar processes are used to obtain the shape feature of each pathology image; the Fourier descriptors of the contour of each cell nucleus are input to the BoW and TF–IDF model to obtain the shape features of the corresponding pathology images. Both the texture features and shape features are limited to 50 dimensions to facilitate the subsequent feature fusion process.

A total of 30 orientations and eight pixels in each cell block were found to be the most suitable for the classification of pathology images after multiple evaluation experiments. In addition, the HOG features are not normalized because the normalization is used to eliminate the difference in contrast due to illumination in the processing of natural images, which is not suitable for medical images. Because of the different sizes of the cavities, the number of cell blocks in each cavity are different. We use the BoW and TF–IDF model to obtain the statistical information of the cell blocks in the cavity, which are selected as the cavity features. The detailed extraction of cavity features is shown in **Figure 2B**.

SURF finds almost as many key points as SIFT, but its vector dimensions are smaller (64 vs 128 dimensions). In addition, ORB is an enhanced method that is based on the features from accelerated segment test (FAST) algorithm, which is the approach that has the lowest dimensionality (32 dimensions) of the extracted features and the least number of feature points. The feature points for SIFT, SURF, and ORB are input to the BoW and TF-IDF model to obtain 50-dimensional feature vectors, as illustrated in **Figure 2C**. As a result, we identified three eigenvectors, all of which represent distinct local aspects of the pathology images as a whole. This is the same as the above features of both the cell nuclei and cavities; they are all concerned with the local area of the image. The pathology images do not have sufficient global features, but the color features and DL features address this deficiency. This makes it possible to optimize the feature fusion.

The color features were designed to consist of 50 dimensions, which is the same as the number of dimensions of other features, to appropriately match in the proposed CNN model. We quantize the pathology images into ten colors in the red, green, and blue (RGB) space, and the distance vectors representing the different distances in which the color distribution is calculated were set as 1, 3, 5, 7, and 9. Thus, a final color feature with 50 dimensions is obtained, as shown in **Figure 2D**.

##### 2.4.1.2 DL feature extraction

**Figure 1B** presents the extraction procedure of the DL features. Because the dataset of pathology images is small, models with multiple parameters may induce the overfitting phenomenon; thus, ResNet-18, which is a small neural network, was selected as the backbone of the CNN. Finally, an integrated network, which includes two convolutional blocks, is used to resize the DL features to the size of the other hand-crafted features.

#### 2.4.2 Matching network

Before the different features are fused, the complex features should undergo some preliminary processing to ensure that the dimensions are the same.

The same hand-crafted features of each pathology image are placed vertically to create a larger set of features. Then, we employ the BoW model in combination with the TF–IDF model to extract a specific low-dimensional set from the single-scale image. For the unsupervised grouping of a large number of extracted features, the BoW method uses the K-means clustering method. In K-means, features that are similar to each other are grouped into a category, aka “clustering”. The TF–IDF uses the term frequency and inverse document frequency to determine the weight vector of features. The term frequency is the number of times a feature appears in all features, and the inverse document frequency measures the uniqueness of a feature.

**Figure 3:**
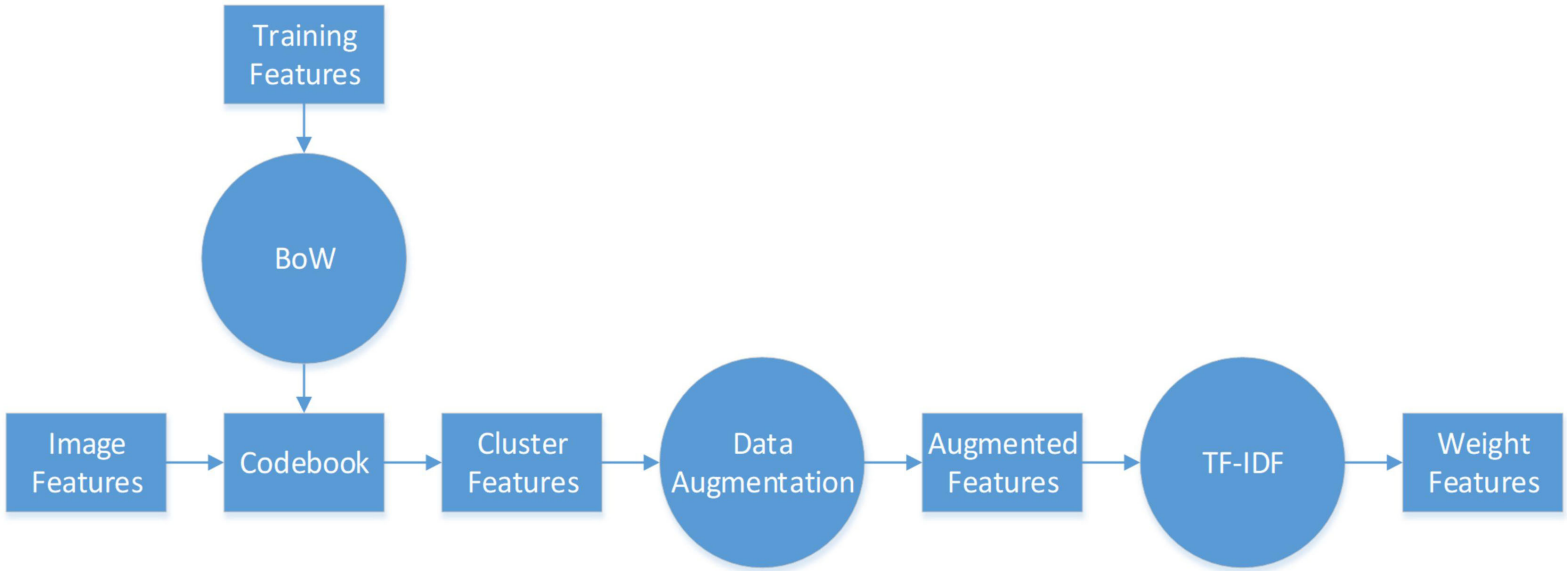
BoW and TF–IDF model.

The flowchart of feature pre-processing is presented in **Figure 3**. First, we use the training feature sets to generate the BoW codebook. The cluster features are the number of times each image feature in the codebook appears in the image. Then, we use augmentation algorithms to add more data to the small datasets to generalize them (see **Algorithm 1**, **2**).

In particular, **Algorithm 1** is used on the cluster features processed by the BoW model, and **2** is used to improve the color features calculated directly from the image. Here, m and n are respectively the dimensionality and augmented multiples of one feature vector. The codebook is used to determine the clustering information for each feature point. Then, we use the normally distributed noise called “perturbation noise” to perturb the clustering information for data augmentation. Lastly, we use the TF–IDF model’s weighting of features to count how often each feature vector occurs in the augmented feature sets.

The operations described above yield one-dimensional features at least 1×50 in size, which is not compatible with the dimensions of the DL features. The one-dimensional feature is a combination of all the hand-crafted features. Therefore, a matching network is necessary to minimize the discrepancies between the sizes of the hand-crafted and DL features, i.e., a 1D-to-2D vector conversion is performed.

The matching network consists of nine convolution blocks, where each one contains fractional strides, batch normalization, and ReLU activation. **Figure 1C** presents the network architecture. The one-dimensional vector with a size of 1×1×50 is converted into a two-dimensional vector with a size of 224×224×3. This is the same as the usual input size of a CNN (except for ResNet (35), VGG-Net (36), and Inception), and the two-dimensional space of DL features. This makes it easier to tune hyperparameters and combine hand-crafted and DL features.

#### 2.4.3 Integrated network

A network is needed to combine the hand-crafted and DL feature vectors. The integrated network consists of a backbone and an additional network. The ResNet, VGG-Net, DenseNet (37) architectures among others were employed as the backbone. As illustrated in **Figure 1D**, the additional network features two convolutional operations and one average pooling layer. The convolutional operation is used to refine the backbone’s output properties by mapping them into low-dimensional space. In addition, the average pooling helps prevent overfitting. We obtain one-dimensional feature vectors for the fusion network.

#### 2.4.4 Fusion network

After the data have been processed by the integrated network, all image features, both hand-crafted and DL features, are converted into one-dimensional vectors with a length of 128. The hand-crafted and DL features are correlated, and hence an early fusion approach that merges various vectors at the feature level is employed to integrate the eight image features before the final classification of the diagnostic pathology.

**Figure 1E** or 1F shows the fusion network architecture. The fusion network has a number of parameters that are unique to it. From left to right, the lengths of each block are 128, 84, 42, and 2. For each feature, as the input vectors decrease, we employ a dropout rate of 0.5 in our fully connected layers. In **Figure 1E**, each hand-crafted feature is joined with the DL feature with the same dimension. This enables the early fusion of different feature vectors. Lastly, the prediction vectors with positive and negative probability values for the above eight features are stacked vertically to create a vector of length 16. Then, after inputting the cascaded one-dimensional vector to the fully connected layer, we predict whether the image is normal or abnormal.

The concatenation of the individual features in the fully connected layer is shown in **Figure 1E**, and **Figure 1F** presents the alternative fusion network, which does not include the concatenation in the fully connected layers.

### 2.5 Fusion CNN implementation

The feature extraction and DCNN models were both implemented using our in-house software. The running environment was Pytorch 1.8.0, CUDA 11.1, and Python 3.7.1 on Windows 10 operating system with an advanced hardware configuration in terms of the GPU and CPU, i.e., GeForce RTX 3090 and Intel Xeon W-2255, respectively. In terms of training parameters, the optimizer was stochastic gradient descent (SGD) with a momentum of 0.9 and weight decay of 0.001, the learning rate was set to 0.01, which decayed by 0.1 every 7 epochs, and the loss function was a cross-entropy loss function describing the distance between two probability distributions. The batch size of each block is listed in **Figure 1C** or **1D**.

## 3 Results and discussion

### 3.1 Data augmentation for hand-crafted features

We used **Algorithm 2** to augment the color features, whereas **Algorithm 1** was used to augment the other hand-crafted features (called “proposed augmentation”). Data augmentation was also performed directly on the images, e.g., rotation, translation, normalization, cropping (called “image augmentation”). After augmentation, we built a database to evaluate our method.

**Figure 4:**
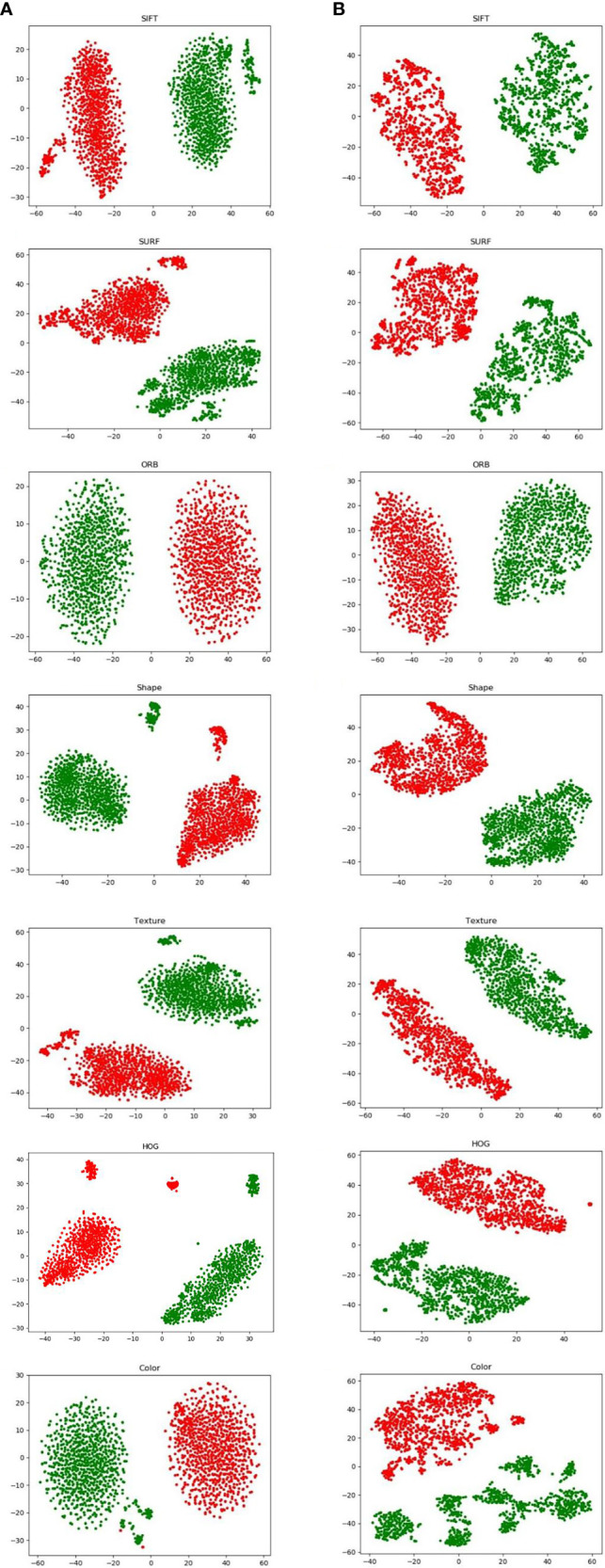
Visualization of hand-crafted features using t-SNE from proposed augmentation **(A)** and image augmentation **(B)**.

**Figure 4** visualizes both augmentation techniques using t-distributed stochastic neighbor embedding (t-SNE). In **Figures 4(B1)–(B7)**, which visualize the results after image augmentation, each class’s data distribution has substantial gaps and overlaps. This shows that augmented pathological image segments do not represent the actual data distribution. In contrast, for the results after proposed augmentation in **Figures 4(A1)–(A7)**, these outliers show that freshly created feature points may fill the gaps left by missing spatial data, which is in line with previous results from image augmentation, but the red and green points from proposed augmentation are more uniformly distributed in the feature space.

**Algorithm 1:** 

**Algorithm 1: **Algorithm 1: Data Augmentation Method 1 **Input:** Cluster feature set: $C=\left\{c_{1},c_{2},\cdots ,c_{m}\right\}$ **Output:** Augmented feature set: $A=\left\{a_{11},\cdots ,a_{1n},a_{21},\cdots ,a_{2n},\cdots ,a_{m1},\cdots ,a_{mn}\right\}$ **for** **do** i∈[1,2,⋯,n] Initialize $g_{ij}=\left\{\right\}$ **for** **do** j∈[1,2,⋯,m] $g_{ij}\cup \left\lceil temp\right\rceil \to g_{ij},\forall temp∼N\left(0,1\right)$ end $G_{i}=g_{ij}+C$ End $C\cup \cup_{i=1}^{n}G_{i}\to A$

**Algorithm 2:** 

**Algorithm 2: **Algorithm 1: Data Augmentation Method 2 **Input:** Cluster feature set: $C=\left\{c_{1},c_{2},\cdots ,c_{m}\right\}$ **Output:** Augmented feature set: $A=\left\{a_{11},\cdots ,a_{1n},a_{21},\cdots ,a_{2n},\cdots ,a_{m1},\cdots ,a_{mn}\right\}$ Initialize G={} **for** **do** i∈[1,2,⋯,n] ${G^{\prime }}_{i}=\left\{g_{1},g_{2},\cdots g_{m}\right\}∼N\left(0,1\right)$ $G_{i}=C+{G^{\prime }}_{i}$ *G*_*i*_=*f*(*G*_*i*_) , where $f=\left|\frac{1}{1+e^{-x}}\right|-0.5$ end end $C\cup \cup_{i=1}^{n}G_{i}\to A$

**Table 1** also presents the results of pathology classification with the proposed augmentation for better understanding. In terms of image accuracy, specificity, and the area under the receiver operating characteristic curve (AUC), the proposed augmentation performs marginally better than the conventional image augmentation; however, the sensitivity of both approaches is equal to 0.9727. Hence, the proposed method is better than augmenting the images directly.

**Table 1:** Performance comparison between proposed augmentation and image augmentation.

Augmentation Method	Accuracy	Specificity	Sensitivity	AUC
Proposed Augmentation	0.9545	0.9364	0.9727	0.9834
					Image Augmentation	0.9555	0.9182	0.9727	0.9783

### 3.2 Performance of the proposed network with different backbones

For the three common deep CNNs (DCNNs) that act as the backbone, we chose ResNet (35), VGG (36), and DenseNet (37). To train each DCNN and produce models of various sizes, a small dataset was input to the three major networks, each of which had different numbers of layers. These networks were ResNet (with 18 or 50 layers), VGG (with 11 or 16 layers), and DenseNet (with 121or 201 layers).

**Table 2** lists the number of parameters of each of the chosen DCNN models as well as the accuracy of the predictions for the training, validation, and test sets. Since there are 546 positive and 554 negative pathology images in the entire dataset of 1100 images, the divided ratio of each sub-dataset for the training, validation, and test sets is 3:1:1. There are strong correlations between the size of the DL model and the size of the data samples. This resulted in either underfitting or overfitting, depending on whether the DL model was too basic or too complicated to provide accurate predictions for the unrelated characteristics included in the small dataset. For the same backbone, there is a tendency for the accuracy to decrease with an increase in the number of parameters, as shown in **Table 2**. Moreover, the best training performance was achieved by ResNet. In particular, ResNet-18 obtained the best test accuracy of 95.45% when compared with the test accuracies of the other DL models.

**Table 2:** Correlation between model size and training accuracy.

Backbone	Parameters	Training Accuracy (%)	Validation Accuracy (%)	Test Accuracy (%)
ResNet-18	14,677,072	99.22	100.00	95.45
ResNet-50	30,547,536	99.46	100.00	94.09
VGG-11	12,721,040	98.80	98.63	93.64
VGG-16	18,215,248	97.35	97.73	93.64
DenseNet-121	11,634,064	99.48	90.00	88.64
DenseNet-201	18,639,248	98.18	90.45	85.91

**Figure 5:**
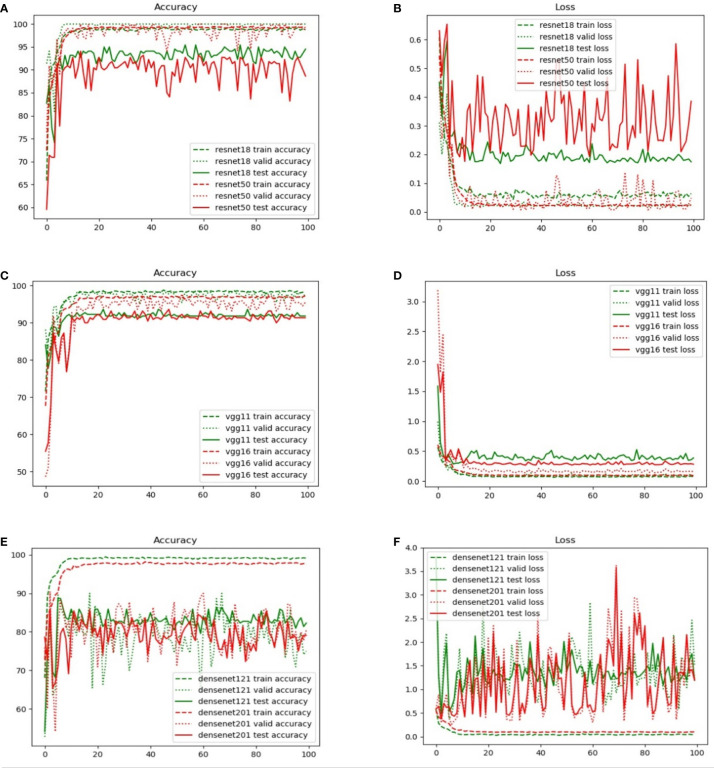
Comparison of the training, validation, and test set accuracy and loss curves with various backbone models: **(A)** ResNet-18, **(B)** ResNet-50, **(C)** VGG-11, **(D)** VGG-16, **(E)** DenseNet-121, and **(F)** DenseNet-201.

**Figure 5** compares the results obtained from different backbone models in terms of the accuracy and loss curves. After epoch 10, the accuracy curves level off, and the loss curves approach equilibrium with only small fluctuations. In particular, the test loss curve of ResNet-50 is more unstable than the loss curve of ResNet-18. This indicates that the accuracy curve of ResNet-50 is less stable than the accuracy curve of ResNet-18. Moreover, because of the small number of parameters, VGG-11 and VGG-16 have variable accuracy performance. Although DenseNet yields the expected outcome in training accuracy, the validation and test accuracies are poor given the excessive number of network layers of DenseNet. This indicates that the model has the problem of overfitting, and the more the layers of the network, the more obvious is the overfitting phenomenon. Furthermore, ResNet exhibits the best performance when compared with VGG and DenseNet. The evaluation indicators, namely, the accuracy, specificity, sensitivity, and AUC, are presented in **Table 3**.

**Table 3:** Performance of the proposed network with different backbones..

Backbone	Accuracy	Specificity	Sensitivity	AUC
ResNet-18	0.9545	0.9364	0.9727	0.9834
ResNet-50	0.9409	0.9636	0.9182	0.9860
VGG-11	0.9364	0.9273	0.9455	0.9639
VGG-16	0.9364	0.9182	0.9545	0.9576
DenseNet-121	0.8864	0.9364	0.8864	0.9483
DenseNet-201	0.8591	0.9000	0.8182	0.9293

**Table 3** lists four quantitative indicators that reveal how well 220 pathology test images (20% of the total images) can be predicted. A total of 110 positive and 110 negative pathology images were analyzed. This performance is based on the proportion of positive and negative samples that were correctly predicted using the accuracy, specificity, sensitivity, and AUC indicators. The proposed model with the ResNet backbone yielded the best performance. In particular, ResNet-18 obtained the best accuracy of 95.45%. This accuracy was higher than those of other models by 1–10 percentage points. The proposed model using ResNet-50 exhibited the expected performance. The highest value of specificity represents that the performance was superior in predicting the negative samples. In particular, ResNet-18 specializes in predicting positive samples, whereas ResNet-50 demonstrates a high performance in predicting negative samples. The nearly equal AUC values of both models demonstrate an equivalent performance in positive and negative prediction. In addition, there are several significant differences between VGG-11 and VGG-16. Both demonstrated acceptable performances (with an accuracy of 93.64%) when compared with the proposed model with a DenseNet backbone. Moreover, an undesirable performance was obtained by DenseNet, and accuracies lower than 90% do not guarantee the accurate prediction of pathology images. This result can be mainly attributed to the poor sensitivity (i.e., a low prediction performance for positive samples), namely, 88.64% for DenseNet-121 and 81.82% for DenseNet-201.

**Figure 6:**
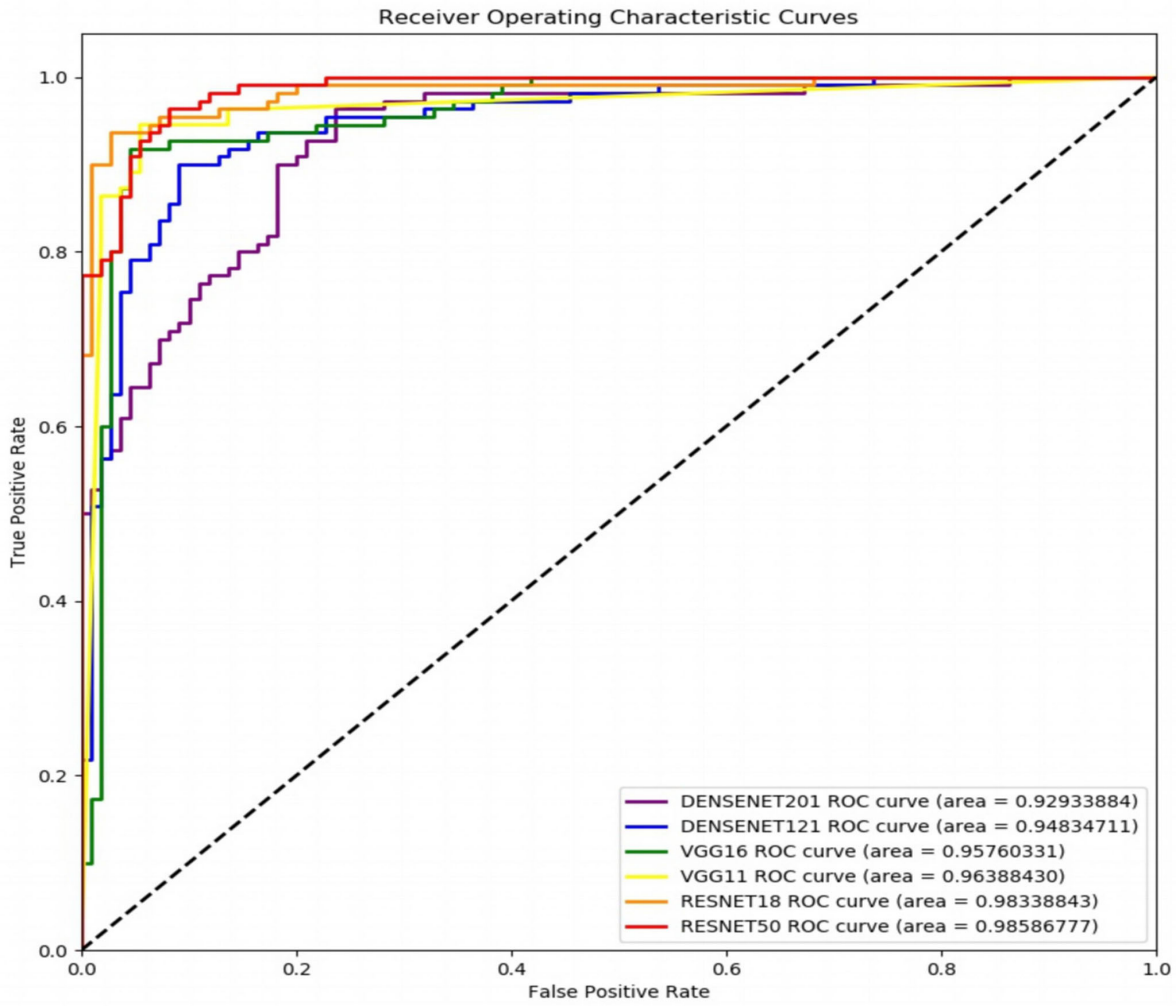
Receiver operating characteristic curves of the proposed network with ResNet-18, ResNet-50, VGG-11, VGG-16, DenseNet-121, and DenseNet-201 backbones.

**Figure 7:**
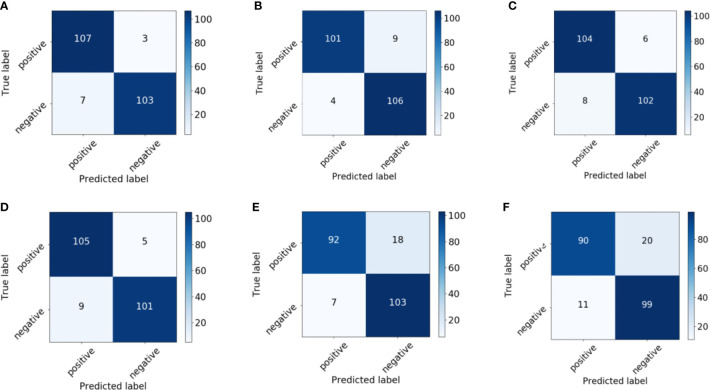
Confusion matrix of the proposed network with **(A)** ResNet-18, **(B)** ResNet-50, **(C)** VGG-11, **(D)** VGG-16, **(E)** DenseNet-121, and **(F)** DenseNet-201 backbones.

**Figure 6** shows the receiver operating characteristic (ROC) curves and AUCs for the proposed model with different backbones, where the specificity and sensitivity are expressed as the false positive rate and true positive rate, respectively. ResNet-18 (orange line) and ResNet-50 (red line) have the highest true positive rate (sensitivity) and lowest false positive rate (specificity), respectively. VGG-11 (yellow line) and VGG-16 (green line) both perform well, although VGG-11 performs somewhat better. The blue and purple lines show that the DenseNets achieve the worst performance. **Figure 7** shows each network’s confusion matrix, which shows the number of correct and incorrect identifications for each category. The 107 true positives for ResNet-18 and 106 true negatives for ResNet-50 show their ability to recognize healthy and diseased tissue. DenseNet-201 has the most false positives (11 samples) and false negatives (20 samples) of all the incorrectly classified samples. DenseNet-121 has a similar performance, which could lead to misdiagnosis in clinical artificial intelligence-assisted therapy.

In general, our recommendation is to use ResNet-18 as the backbone in the proposed deep network for assistance in the diagnosis of prostate cancer pathology images. This is because ResNet-18 has the highest accuracy, is the most stable, and converges to the correct value for the loss function. The following experiments are therefore based on ResNet-18.

### 3.3 Individual features vs fused features

**Figure 8:**
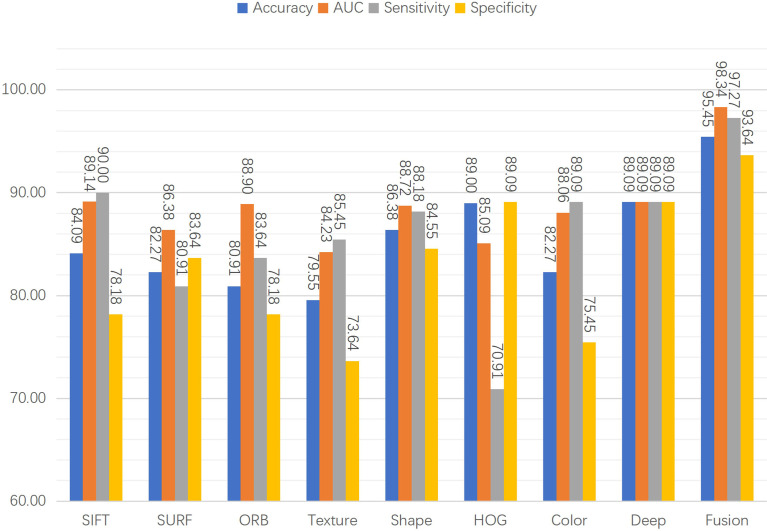
Classification performance with individual features or fused features.

Eight features were extracted from the pathology images to detect prostate cancer. In particular, seven hand-crafted features, namely, the SIFT, SURF, and ORB local features, the texture and shape of the cell nuclei, the HOG of the cavities, color of the image, and the DL feature were extracted. As shown in **Figure 8**, to evaluate the performance of the fused features, we examined the proposed network’s performance using various features. Performance varies based on the distinct qualities of each feature. There is a similarity in that none of the hand-crafted features outperform the DL feature. Moreover, there are significant differences in the accuracy, sensitivity, specificity, and AUC indicators for each feature, and none exceed 90%. The results for the fused features in **Figure 8** demonstrate that the proposed DCNN overcame this constraint by fusing the various hand-crafted and DL features. With the help of the seven hand-crafted features, the DL feature’s accuracy, AUC, sensitivity, and specificity increased from 89.09 (for all indicators) to 95.45, 98.34, 97.27, and 93.64, respectively.

### 3.4 Performance of the fusion network with and without concatenation

This section presents the influence of concatenation on the fusion network, which was also used without concatenation to fuse the seven different hand-crafted features, the DL feature alone, and all fused features.

**Table 4** shows the evaluation results. First, a comparison of the first two rows of **Table 4** shows that the concatenation substantially enhances the classification results in terms of accuracy, specificity, sensitivity, and AUC. Moreover, we compare the results for the fused hand-crafted features, the DL feature, and the fusion of all features using the fusion network without concatenation. The use of the DL feature and fused hand-crafted features significantly improves the classification performance, as shown in the final three rows of **Table 4**. Overall, the fusion of seven hand-crafted features and the DL feature improves the classification results, and the concatenation of the fusion network further improves these results.

**Table 4:** Classification performance with different features and feature fusion with/without concatenation.

Fusion Network	Accuracy	Specificity	Sensitivity	AUC
All Feature Fusion with Concatenation	0.9545	0.9364	0.9727	0.9834
All Feature Fusion without Concatenation	0.9091	0.8727	0.9455	0.9698
Deep Feature	0.8909	0.8909	0.8909	0.8909
Hand-crafted Feature Fusion without Concatenation	0.8636	0.8091	0.9182	0.9036

### 3.5 Ten-fold cross-validation of ResNet-18

**Figure 9:**
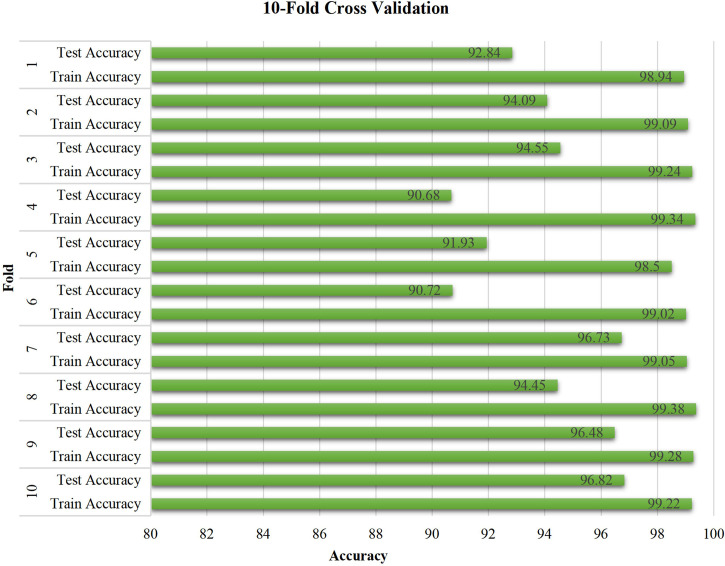
Ten-fold cross validation.

The stability of the proposed model was verified by conducting a 10-fold cross-validation experiment, as shown in **Figure 9**, which reveals the performance of the proposed model with respect to unseen pathology images. The training accuracy is constant at approximately 99%. Moreover, the test accuracy exhibits relatively large fluctuations when compared with the training accuracy. Most test accuracies for each fold are over 94%, especially the three highest values of 96.82%, 96.73%, and 96.48% for the tenth, seventh, and ninth folds, and with the exception of 90.68% and 90.72% for the fourth and sixth folds. Overall, the experimental results demonstrate the effectiveness and stability of the proposed model.

### 3.6 Heatmaps using Grad-CAM++

**Figure 10:**
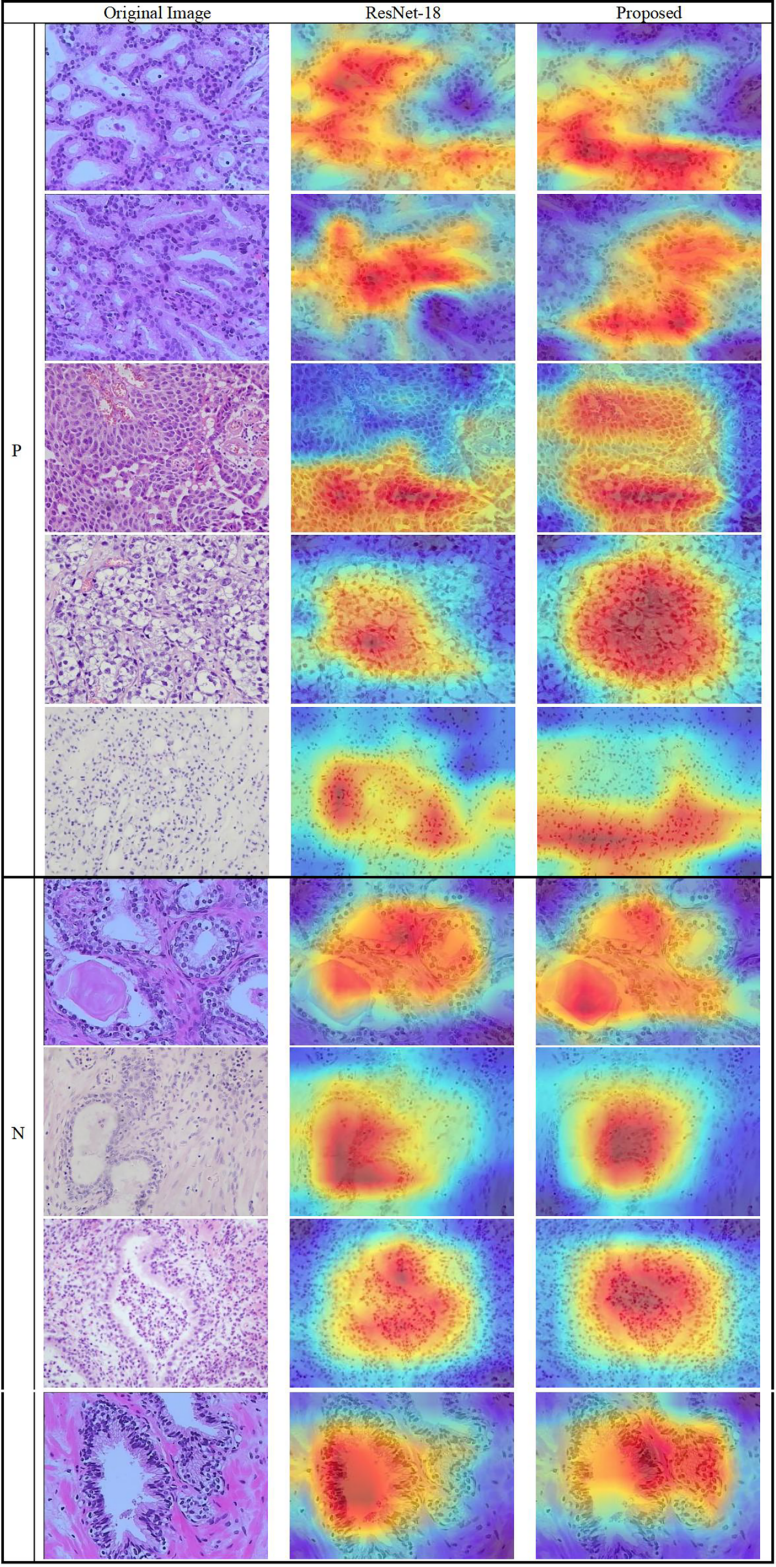
Heatmap of the lesions using Grad-CAM++. (Left column) original images, (middle column) results of ResNet-18, and (right column) results of the proposed method.

The results show that the proposed model’s region of focus in the pathology images differs significantly from that used in ResNet-18’s direct classification. Using Grad-CAM++ (38), a heatmap was created to demonstrate the differences between the two models and is shown in **Figure 10**. The top five rows present samples with positive pathology results, and the bottom four rows present the negative samples. In most cases, we confirmed that the proposed deep learning model classify each class by assigning weights to cell nucleus and glands lesions more appropriately. Although not all actual pathognomonic areas were weighted, the highlighted areas have more prominent nucleoli and larger nuclei, more close and crowded glands, and increased pleomorphism.

## 4 Conclusions

By combining eight image features, a novel DL classification model was created. The eight features consist of the SIFT, SURF, and ORB local image features; the form and texture of cell nuclei; the HOG cavity feature; color; and a CNN DL feature. Among them, the seven hand-crafted features are extracted using a BoW and TF–IDF model along with a novel data augmentation method, and the DL feature is extracted using ResNet-18.

The proposed DL network includes matching, integrated, and fusion networks. A backbone and an additional network comprise the integrated network. The size of the hand-crafted features is converted to the size of the image using the matching network, and the eight two-dimensional vectors are integrated and processed using the integrated network. The fusion network includes eight fully connected layers with concatenation to effectively fuse the distinct properties of each feature and classify the pathology images.

ResNet-18/50, VGG-11/16, and DenseNet-121/201 were evaluated in the experiments. The results show that ResNet-18 obtains the best accuracy, sensitivity, specificity, and AUC. We further evaluated the individual features, the two proposed augmentation methods, the fusion network design, model stability, and model-focused heatmaps to ensure that the proposed network employed the best design. The suggested method could be suitable for other pathology images, such as those of breast cancer and thyroid cancer, because it is based on the underlying principles of feature engineering and DL.

## Data availability statement

The raw data supporting the conclusions of this article will be made available by the authors, without undue reservation.

## Ethics statement

The studies involving human participants were reviewed and approved by the ethics committee of biological and medical research of Peking University and Guangdong Provincial People’s Hospital, China. Written informed consent for participation was not required for this study in accordance with the national legislation and the institutional requirements.

## Author contributions

XH contributed to collect and analyse data, manuscript writing. ZL contributed to analyse data and manuscript writing. MZ contributed to collect data and pathology diagnosis. SG contributed to manage and coordinate this project. All authors contributed to the article and approved the submitted version.

## Funding

This work was supported by the National Natural Science Foundation of China (Grant No. 61901008 and 12075011), Beijing Municipal Natural Science Foundation of China (Grant No. 7202093), and Key Research and Development Program of Science and Technology Planning Project of Tibet Autonomous Region, China (Grant No. XZ202001ZY0005G).

## Acknowledgments

We thank the help of technicians from Department of Pathology in Peking University Health Science Center and in Guangdong Provincial People’s Hospital, China.

## Conflict of interest

The authors declare that the research was conducted in the absence of any commercial or financial relationships that could be construed as a potential conflict of interest.

## Publisher’s note

All claims expressed in this article are solely those of the authors and do not necessarily represent those of their affiliated organizations, or those of the publisher, the editors and the reviewers. Any product that may be evaluated in this article, or claim that may be made by its manufacturer, is not guaranteed or endorsed by the publisher.
